# Advanced Manufacturing Routes for Electrodes and Cells in All‐Solid‐State Batteries: From Powder to Power

**DOI:** 10.1002/smtd.70922

**Published:** 2026-08-03

**Authors:** Heejoo Park, Soyeon Kong, Jihyun Jang

**Affiliations:** ^1^ Department of Chemistry Sogang University Seoul Republic of Korea; ^2^ Center for Nano Materials Sogang University Seoul Republic of Korea

**Keywords:** all‐solid‐state batteries, cell fabrication, cell stacking, dry‐process, electrode fabrication, wet‐process

## Abstract

All‐solid‐state batteries (ASSBs) represent promising next‐generation energy storage systems with superior safety and energy density compared to conventional lithium‐ion batteries (LIBs). This review comprehensively examines advanced electrode and cell manufacturing processes that are critical to the commercialization of ASSBs. Electrode fabrication processes are categorized into wet and dry processing approaches. The wet processing leverages existing LIB manufacturing infrastructure for cost‐effectiveness but faces challenges due to chemical reactivity between sulfide solid‐state electrolytes and processing solvents/binders. Conversely, dry processing offers shorter production steps and improved environmental sustainability, particularly for thick electrodes. For cell assembly, bipolar stacking architectures enhance volumetric energy density through internal series connectivity, while Z‐folding methodology enables scalable manufacturing. Pressure optimization emerges as crucial for maintaining interfacial contact and electrochemical performance. The manufacturing strategies presented provide essential insights for transitioning ASSB technology from laboratory to commercial production, addressing technical and economic barriers to widespread adoption.

## Graphical abstract

All‐solid‐state battery manufacturing requires coordinated advances in wet processing, dry processing, pressurization, and cell stacking. This review summarizes scalable routes for fabricating ASSB components and integrating them into practical cell architectures, highlighting how solvent compatibility, binder design, pressure control, and bipolar stacking govern the transition from laboratory‐scale cells to commercial production.

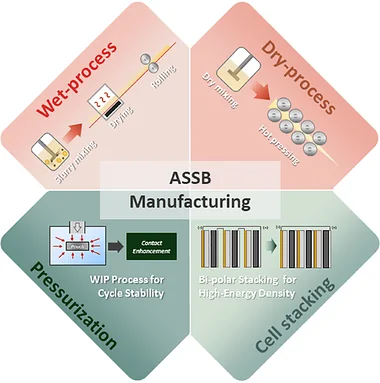

## 1 Introduction

**FIGURE 1:**
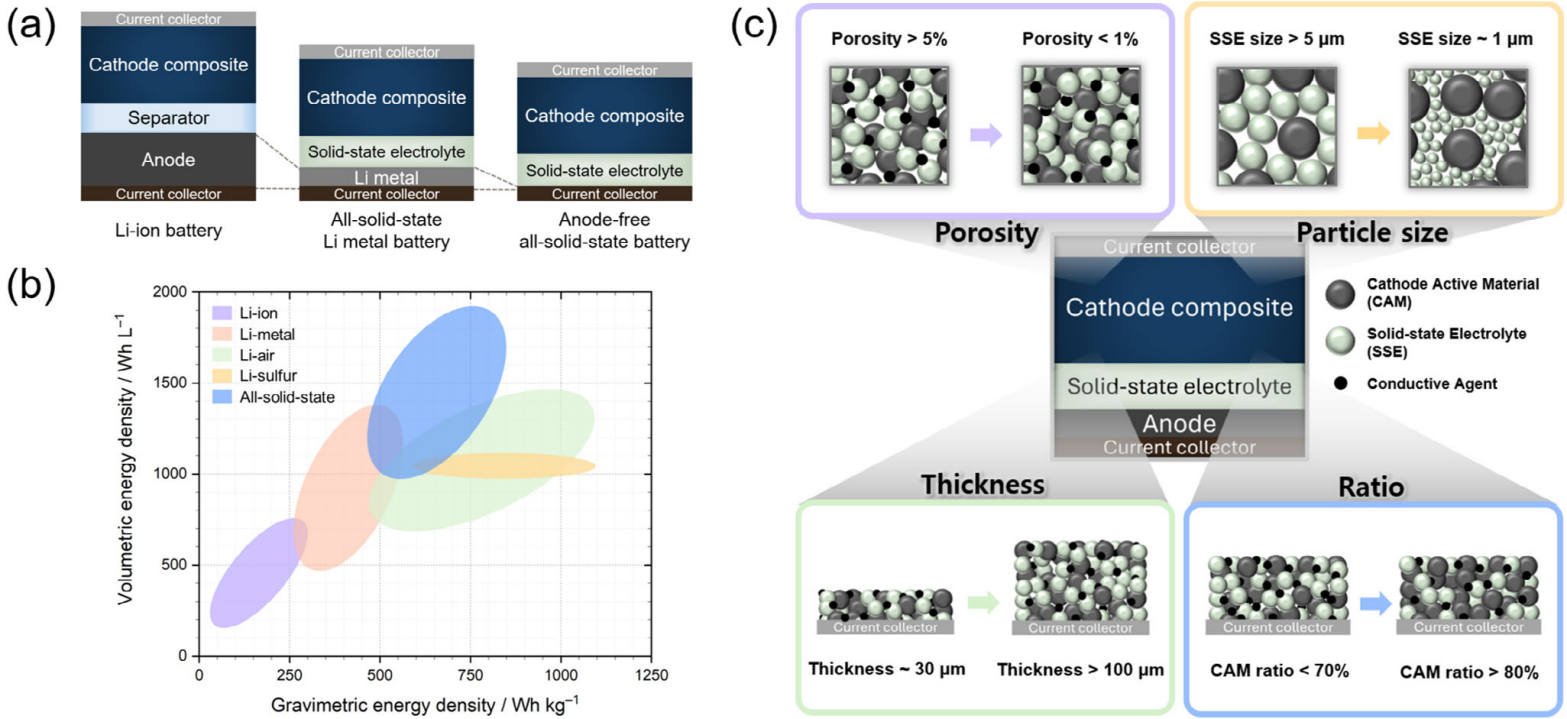
Necessity and advantages of ASSBs in light of architectural factors. (a) Schematic representation of conventional LIBs, all‐solid‐state lithium metal batteries, and anode‐free ASSBs. (b) Relationship between volumetric and gravimetric energy density for different batteries. Reproduced with permission [6]. Copyright 2019, IOP Publishing Ltd. (c) Effects of architectural parameters on various electrode properties.

Commercial lithium‐ion batteries (LIBs) with organic liquid electrolytes provide reliable energy storage for portable electronics, electric vehicles, and other mobile devices [1, 2]. However, the increasing demand for enhanced safety and higher energy density has driven the development of all‐solid‐state batteries (ASSBs), which replace flammable liquid electrolytes with solid‐state electrolytes (SSEs) and are considered among the most promising next‐generation batteries [3, 4]. To accurately estimate practical energy densities, it is essential to account for all inactive components (SSEs, electrode additives, metal foils) and consider the proper capacity limits of the active material [5]. Figure 1a depicts the typical schematic representation of LIBs and ASSBs with a change in energy density upon replacing the liquid electrolyte with an SSE. The development of ASSBs using SSEs enhances thermal and chemical safety and opens the potential for next‐generation systems with significantly higher energy densities, such as those employing pure silicon anodes, lithium‐metal anodes, and anode‐free designs (Figure 1b) [6, 7]. Nevertheless, several critical challenges remain, including high interfacial resistance between electrodes and SSEs, mechanical instability, and the inherently low ionic conductivity of SSEs. To address these limitations, extensive efforts have been devoted to developing SSEs as core components of ASSBs. Recent studies have further broadened the design criteria for SSEs beyond ionic conductivity alone. Sulfide/polymer composite and solid polymer electrolytes have been developed to balance ionic conductivity, mechanical flexibility, interfacial stability, and processability, while enabling thinner electrolyte configurations [8, 9]. Emerging phase‐modulation strategies, including dielectric and liquid‐crystal phases, also suggest new routes to regulate ion transport and interfacial behavior through tailored polymer‐phase architectures [10]. In addition, halide‐based SSEs have attracted increasing attention as manufacturable candidates for ASSB pilot lines because of their high‐voltage stability, moderate‐to‐high ionic conductivity, and compatibility with scalable processing [11].

The energy density and performance of ASSBs are highly dependent on architectural parameters, such as porosity, particle size, thickness, and the cathode composite ratio (Figure 1c) [12]. Unlike in liquid systems, voids remain unfilled and act as barriers to ion transport, lowering volumetric energy density and mechanical stability and bottlenecking kinetic performances [13]. The particle size of both the active material and solid electrolyte also plays a decisive role by controlling solid–solid contact areas, percolation networks, and tortuosity [14]. Small particles provide a large contact area but carry a higher risk of interfacial reactivity, whereas larger particles stabilize the surface but reduce active contact. Bimodal or trimodal size distributions have been shown to improve packing density and connectivity [15, 16]. Electrode thickness further introduces trade‐offs; thicker layers enhance areal capacity but intensify ionic resistance and reaction inhomogeneity [17, 18, 19]. The composition ratio among active material, solid‐state electrolyte, and conductive additive defines the balance between ionic/electronic conductivity and energy density. Higher active material loadings increase the capacity, but inadequate electrolyte or carbon pathways can severely limit the transport of ions and electrons [20]. Therefore, the commercialization of ASSBs requires optimized electrode architectures and manufacturing processes that simultaneously ensure safety and maximize energy density [21, 22].

Based on these characteristics in electrode manufacturing processes, additional critical considerations arise during cell assembly using the fabricated electrodes. In ASSB systems, the interface between electrodes and SSE cannot achieve sufficient ionic conductivity through simple physical contact alone, necessitating continuous external pressure [23, 24, 25, 26]. This is not merely a temporary requirement during the manufacturing process, but a critical operating condition that must be maintained throughout battery operation [27, 28, 29]. Interestingly, this pressure dependency enables innovative cell structure designs unique to ASSBs. In ASSB systems where there is no risk of liquid electrolyte leakage, bipolar stacking structures can be implemented, where both cathode and anode are located on opposite sides of a single current collector, dramatically improving cell voltage with series connection and energy density by reducing the number of current collectors as well as minimizing internal resistance [30, 31, 32]. This review systematically examines these comprehensive electrode and cell fabrication processes.

## 2 Types of Electrodes

**FIGURE 2:**
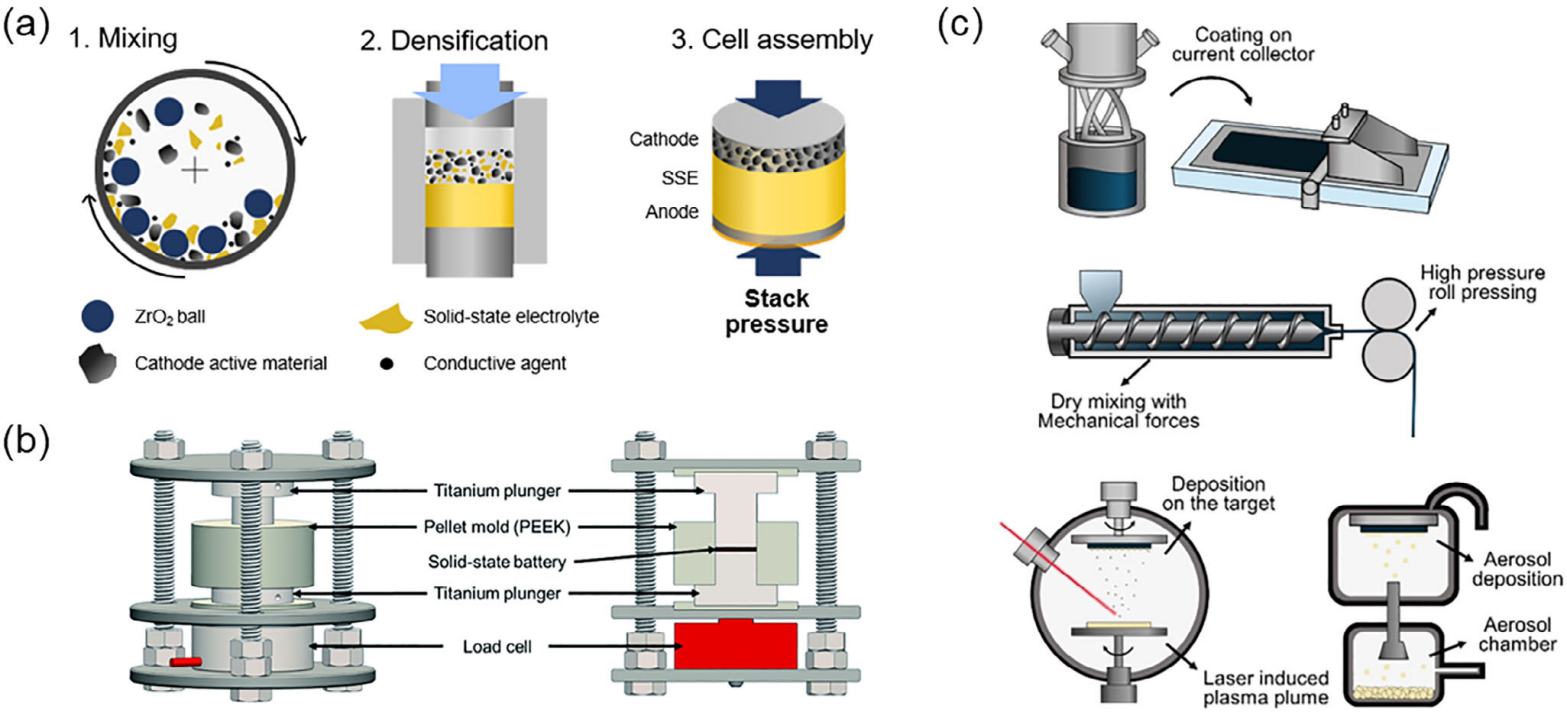
(a) Schematic illustration of the fabrication process for pellet‐type cell: mixing, densification, and cell assembly. Adapted with permission [33]. Copyright 2021, ACS Publications. (b) Design of the ASSB holder with a load cell to monitor the stack pressure during cycling and conductivity measurements. Reproduced with permission [22]. Copyright 2020, Royal Society of Chemistry. (c) Electrode fabrication methods for sheet‐type electrodes: slurry coating process, dry film process, pulsed laser deposition, and aerosol deposition method. Reproduced with permission [43]. Copyright 2021, ACS Publications.

In the laboratory‐scale fabrication stage, electrode fabrication for ASSBs can generally be classified into two main types: pellet‐type and sheet‐type electrodes [21]. The pellet‐type electrode is produced by compacting the powdered electrode components into a dense pellet, typically several hundred micrometers thick, under high pressure. As illustrated in Figure 2a, the electrode components are first mixed to prepare the cathode composite, after which the SSE powder is placed into a polyaryletheretherketone (PEEK) mold and pressed to form an SSE pellet [33]. The cathode and anode composites are then sequentially placed on each side of the SSE pellet and densified under pressure. Subsequently, stainless steel or titanium metal rods (plungers) are positioned on both ends to serve as current collectors. Figure 2b shows the overall design of the holder used for operating a pellet‐type cell [26]. The holder comprises a PEEK housing and upper and lower plungers, with the pellet cell sandwiched between the plungers. Mechanical pressure is applied using a tightening jig, and a load cell is incorporated to monitor stack pressure during electrochemical cycling [21]. In this configuration, the SSE layer is essential for the mechanical stability of the cell and must therefore be sufficiently thick; however, a thicker SSE layer is detrimental to the energy density of the cell [34, 35]. Furthermore, the pelletizing process is generally considered unsuitable for large‐scale production because of its time‐ and cost‐intensive nature. Therefore, pellet‐type electrodes are primarily used in early research, whereas sheet‐type electrodes are better suited for mass production [36].

Sheet‐type electrodes are considered a practical platform for scaling up ASSBs because they are compatible with continuous manufacturing and allow better control over electrode thickness, morphology, and areal loading [37, 38]. As shown in Figure 2c, various fabrication routes have been explored for sheet‐type electrodes, including slurry coating, dry film processing, pulsed laser deposition, and aerosol deposition [39, 40, 41, 42, 43]. Among these methods, slurry coating is the most similar to conventional LIB manufacturing and can potentially utilize existing production infrastructure. This process involves preparing an electrode slurry and casting it as a thin film on a current collector. Although slurry coating is fast, scalable, and suitable for thickness control [44], careful optimization of slurry composition is required because binder–solvent compatibility and the chemical stability of SSEs strongly affect slurry properties and electrode microstructure [45]. These challenges have also motivated the development of dry film processing and other solvent‐minimized fabrication routes for sheet‐type ASSB electrodes.

## 3 Wet Process for Sheet‐type ASSB Components

### 3.1 Selection of Solvent

In conventional LIB production, slurry casting is widely adopted for its compatibility with large‐scale manufacturing. However, when this wet process is applied to ASSBs, the chemical reactivity between sulfide‐based SSEs and slurry components, such as solvents and binders, presents significant challenges [45]. In particular, solvent selection is critical because sulfide SSEs are highly sensitive to solvent polarity and donor characteristics, which can lead to electrolyte structural degradation and deterioration in electrochemical performance [40, 42].

**FIGURE 3:**
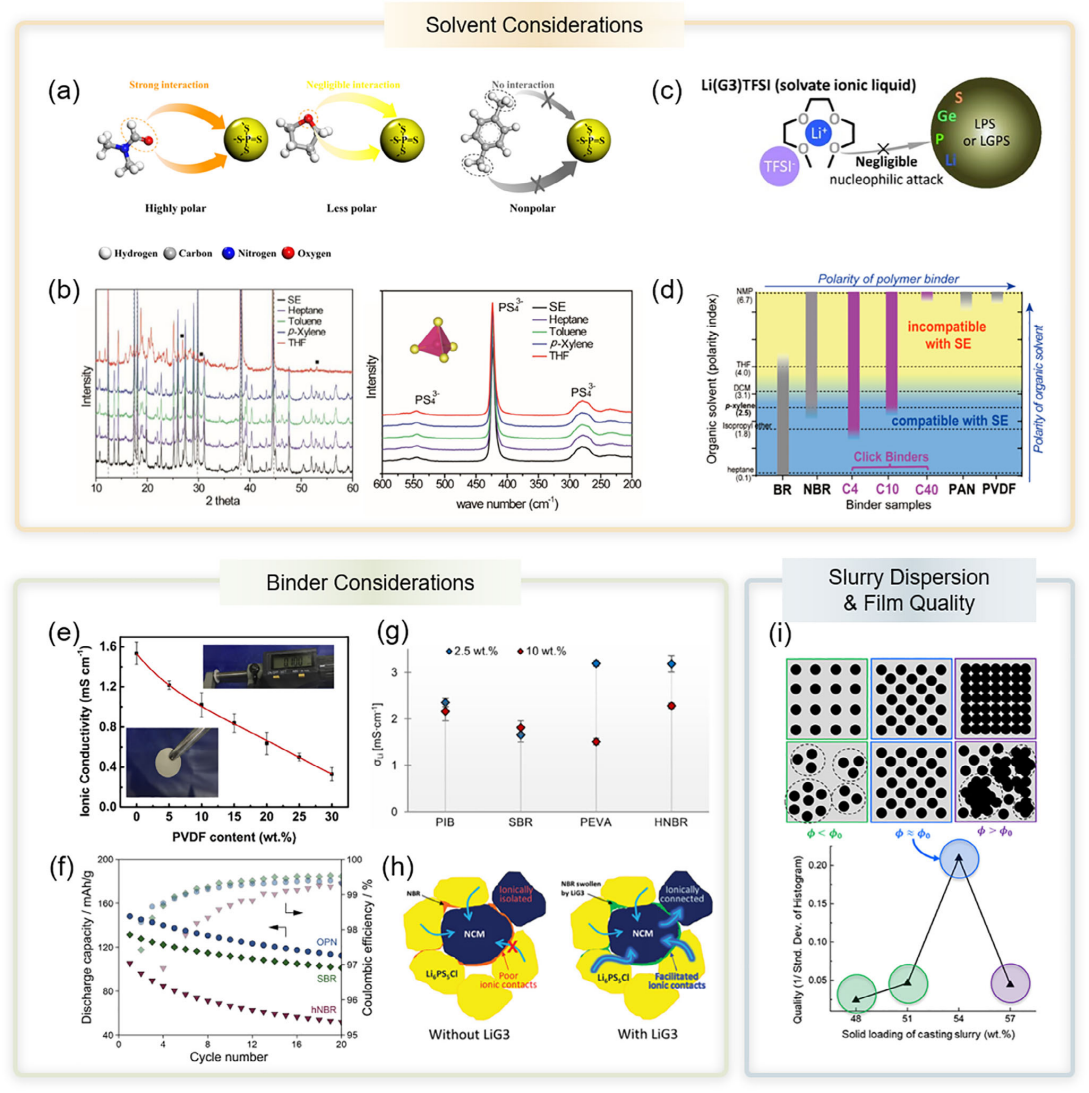
(a–d) Compatibility considerations for selecting wet solvents. (a) Schematic illustrating interactions between lithium thiophosphates and solvents with various polarities. Reproduced with permission [46]. Copyright 2024, Springer Nature. (b) The XRD patterns and Raman spectra of the SSE after immersing in various organic solvents for 24 h. Reproduced with permission [48]. Copyright 2017, IOP Publishing Ltd. (c) Schematic diagram of reactivity for glyme‐based liquids and the composite electrode microstructure, showing that LiG3 improves imperfect solid–solid contacts. Reproduced with permission [49]. Copyright 2024, Wiley. (d) Polarity window showing that binders and SSEs are compatible. Reproduced with permission [50]. Copyright 2019, ACS Publications. (e‐h) Compatibility considerations for selecting binders for wet processing. (e) Ionic conductivities of composite electrolytes at 25°C as a function of PVdF content. Reproduced with permission [51]. Copyright 2021, Wiley. (f) Specific discharge capacities over 20 cycles and corresponding Coulombic efficiencies. Reproduced with permission [52]. Copyright 2020, Elsevier Inc. (g) Estimated ionic conductivity for calendared SE sheets containing PIB, SBR, PEVA, and hNBR binders at 30°C. Reproduced with permission [53]. Copyright 2018, IOP Publishing Ltd. (h) Schematic diagram illustrating microstructures of NCM electrodes without and with binder. Reproduced with permission [54]. Copyright 2019, Wiley. (i) Correlation between solid loading, dispersion stability, and the quality of tape‐cast sulfide SSE films. Reproduced with permission [55]. Copyright 2021, Elsevier.

The reactivity of sulfide SSEs is strongly affected by the polarity of the solvent. Li et al. systematically investigated the interactions between lithium thiophosphate electrolytes and solvents of varying polarity (Figure 3a) [46]. Strong decomposition was observed with highly polar solvents such as dimethylformamide, whereas tetrahydrofuran (THF), with intermediate polarity, exhibited only minor interaction. In contrast, nonpolar solvents such as p‐xylene showed virtually no reaction with the SSEs. These results indicate that low‐polarity solvents are generally more suitable for preserving the structural integrity of sulfide electrolytes during slurry processing. Li_3_PS_4_‐based SSEs tend to dissolve or lose crystallinity upon contact with high‐polarity solvents due to nucleophilic attack, particularly at phosphorus sites, which behave as hard acids and are susceptible to hard base donors such as oxygen [47].

A similar trend was confirmed by direct solvent‐immersion tests. The chemical compatibility of 75Li_2_S–25P_2_S_5_ electrolytes with various solvents was evaluated using 24 h immersion followed by X‐ray diffraction (XRD) analysis (Figure 3b) [48]. The original crystalline structure of the SSE was preserved in low‐polarity solvents such as heptane (*p* = 0.1), toluene (*p* = 2.4), and p‐xylene (*p* = 2.5). However, significant structural degradation was observed in THF (*p* = 4.0), including the formation of impurity phases such as Li_2_S [40]. Raman spectroscopy further revealed that while the characteristic vibrations of PS_4_^3–^ units were retained, the overall crystallinity was reduced, indicating that THF disrupts the structural ordering of the electrolyte. These findings show that solvent polarity provides a practical guideline for screening solvents for sulfide‐based slurry processes.

Although ether‐based solvents containing electron‐donating oxygen atoms can induce chemical instability in sulfide SSEs, their reactivity can be suppressed by modifying the solvent coordination environment. Oh et al. reported that solvate ionic liquids formed from G3 (triglyme) and lithium bis(trifluoromethanesulfonyl)imide (LiTFSI) salts significantly reduced the reactivity of ether oxygen atoms with sulfide SSEs (Figure 3c) [49]. In these systems, the donor strength and nucleophilicity of ether oxygen atoms are reduced by complexation with the salt, thereby minimizing their interaction with the SSEs. This salt–solvent coordination structure improves chemical compatibility and facilitates ion transport across the solid–solid interface, as evidenced by the improved capacity of LiFePO_4_‐based composite electrodes.

While low‐polarity solvents are generally preferred to suppress the decomposition of sulfide SSEs, solvent selection cannot be determined solely by SSE stability. For practical slurry processing, the solvent must also dissolve or swell the polymer binder and support sufficient cohesion within the electrode composite. Figure 3d illustrates this solvent‐centered compatibility window by correlating the polarity of organic solvents with the usable polarity range of polymer binders and the chemical stability region of sulfide SSEs [50]. Lee et al. used thiol‐ene click chemistry to tune the polarity of polymeric binders and identify solvent–binder combinations that satisfy the processing requirements for sulfide‐based composite electrodes. Their analysis showed that sulfide electrolytes such as Li_6_PS_5_Cl (LPSCl) are chemically stable in low‐polarity solvents with a polarity index below 2.5, such as p‐xylene, while the applicable binder range is further constrained by the polarity match between the selected solvent and binder [34, 40]. Among the synthesized click‐polymer binders, C4 and C10 were located within the compatibility zone, demonstrating that suitable solvent selection requires balancing SSE stability, binder solubility, and electrode cohesion.

### 3.2 Selection of Binders

In slurry‐based processes for ASSBs, selecting an appropriate binder is critical to ensuring chemical stability with the SSEs and maintaining the mechanical integrity of the electrode [56, 57]. Binders provide adhesion between the solid electrolyte and the active material and directly influence slurry dispersibility and electrode properties. Lee et al. explained that binders used in ASSBs are generally expected to satisfy four key criteria: (i) chemical inertness toward the solid electrolyte, (ii) sufficient solubility in the selected solvent, (iii) strong adhesion to both the solid electrolyte and the current collector, and (iv) minimal loss of ionic conductivity of the solid electrolyte [48].

Binder content is one of the most important parameters because it directly affects the balance between mechanical stability and ionic conductivity. Wang et al. introduced poly(vinylidene fluoride) (PVdF) as a binder into LPSCl using ethyl acetate as the solvent to fabricate thin and flexible SSE sheets (Figure 3e) [51]. As the PVdF content increased, the Li ion conductivity of the electrolyte decreased from 1.21 to 0.33 mS cm^−1^. However, at a thickness of 110 µm, the addition of PVdF effectively suppressed lithium dendrite growth and significantly improved the mechanical stability of the SSE. This result suggests that the binder content should be carefully optimized to provide sufficient mechanical reinforcement while minimizing the loss of ionic conductivity.

Binder chemistry also affects the processability and electrochemical performance of composite cathodes. Teo et al. compared the electrochemical performance and processability of polyisobutene (PIB), styrene‐butadiene rubber (SBR), and hydrogenated nitrile‐butadiene rubber (hNBR) in slurry‐processed cathodes comprising LiNi_0.6_Co_0.2_Mn_0.2_O_2_ (NCM622) and sulfide SSEs (Figure 3f) [52]. PIB exhibited the best electrochemical performance, delivering a discharge capacity of 148 mAh g^−1^ and good process stability. In contrast, hNBR showed the lowest initial capacity and Coulombic efficiency, which was attributed to its chemical reactivity with the solid electrolyte. These results demonstrate that binder selection affects not only slurry processability and mechanical cohesion but also the chemical stability of the composite electrode.

The effect of binder chemistry has also been investigated in SSE sheet fabrication. Riphaus et al. systematically evaluated five binders to fabricate Li_10_SnP_2_S_12_‐based SSE sheets (Figure 3g) [53]. They found that the polarity and molecular structure of the binders significantly influenced processability, mechanical flexibility, porosity, and Li‐ion conductivity of the electrolyte sheets. Among the tested binders, hNBR and poly(ethylene vinyl acetate) (PEVA) exhibited superior surface adhesion, making them suitable for fabricating thin and uniform electrolyte sheets, while PIB provided sufficient flexibility and low resistance even at low binder content. These results indicate that the optimal binder can differ depending on whether the target component is a composite cathode or an SSE sheet.

In addition to conventional binders, ionically conductive binders have been introduced to improve Li^+^ transport within composite electrodes. Figure 3h illustrates a case in which a conductive binder was applied to secure continuous Li^+^ transport pathways in the electrode. Oh et al. fabricated slurry‐based composite cathodes using LPSCl SSE and NCM622 active material by introducing the ionically conductive binder NBR–Li(G3)TFSI [54]. When 3.5 wt.% LiG3 was incorporated, the initial discharge capacity increased to 174 mAh g^−1^, the Coulombic efficiency improved to 90%, and the interfacial resistance was significantly reduced. This improvement was attributed to the ionically conductive binder, which provides continuous Li^+^ pathways (as indicated by the blue arrows in Figure 3h) and effectively connects NCM particles to the LPSCl SSE.

Beyond solvent and binder selection, slurry composition and solid loading are also decisive in determining dispersion stability and final film quality. Solid loading controls the interparticle distance and the balance of interactions within the dispersion, directly affecting slurry stability, film uniformity, and defect formation. Figure 3i illustrates the correlation between solid loading and film quality in sulfide solid electrolyte slurries based on the extended Derjaguin–Landau–Verwey–Overbeek (X‐DLVO) theory, together with image histogram analysis [55]. At the optimized solid loading of 54 wt.%, defect‐free films were obtained, exhibiting a high ionic conductivity of 1.12 mS cm^−1^ and an area‐specific resistance reduced by a factor of 11. This study demonstrates that high‐quality thin films can be produced via tape‐casting processes when slurry dispersion, binder selection, and solid loading are properly controlled, providing useful guidance for process optimization to improve the energy density of ASSBs.

### 3.3 Thickness Control of SSE Sheets

In sheet‐type ASSBs, SSE thickness is a primary lever for cell‐level energy density. However, many laboratory studies still rely on pelletized sulfide membranes with a thickness of ∼800 µm and an area of ∼1.5 cm^2^, which are impractical for high‐energy applications and costly to fabricate [58, 59, 60]. Thinning the SSE reduces inactive mass/volume and areal resistance, motivating a transition from bulky pellets to ultrathin, sheet‐type membranes for commercially relevant cells. Modeling analyses (e.g., SolidPAC) indicate that meeting ∼350 Wh kg^−1^ typically requires LPSCl membranes below ∼60 µm in LiNi_0.8_Co_0.1_Mn_0.1_O_2_ (NCM811) | Li cells, ∼29 µm in LiCoO_2_ (LCO) | Li cells, and ∼80 µm in S | Li cells, with a broader working range of ∼20–80 µm across chemistries; likewise, approaching ∼750 Wh L^−1^ often calls for SSE thicknesses <200 µm, particularly when combined with bipolar stacks [61]. Wet processing offers a practical route to such ultrathin membranes at large area and continuous throughput, provided that solvent–binder systems are selected to maintain chemical integrity and that slurry rheology and drying are tightly controlled for sulfide‐ and halide‐based chemistries [34]. From this standpoint, realizing ultrathin SSEs requires a coordinated set of strategies, including solvent/binder combinations tailored to reactivity windows, lamination or calendering for densification, and mechanical or interfacial reinforcement, to balance thickness, areal conductance, interfacial robustness, and manufacturability.

**FIGURE 4:**
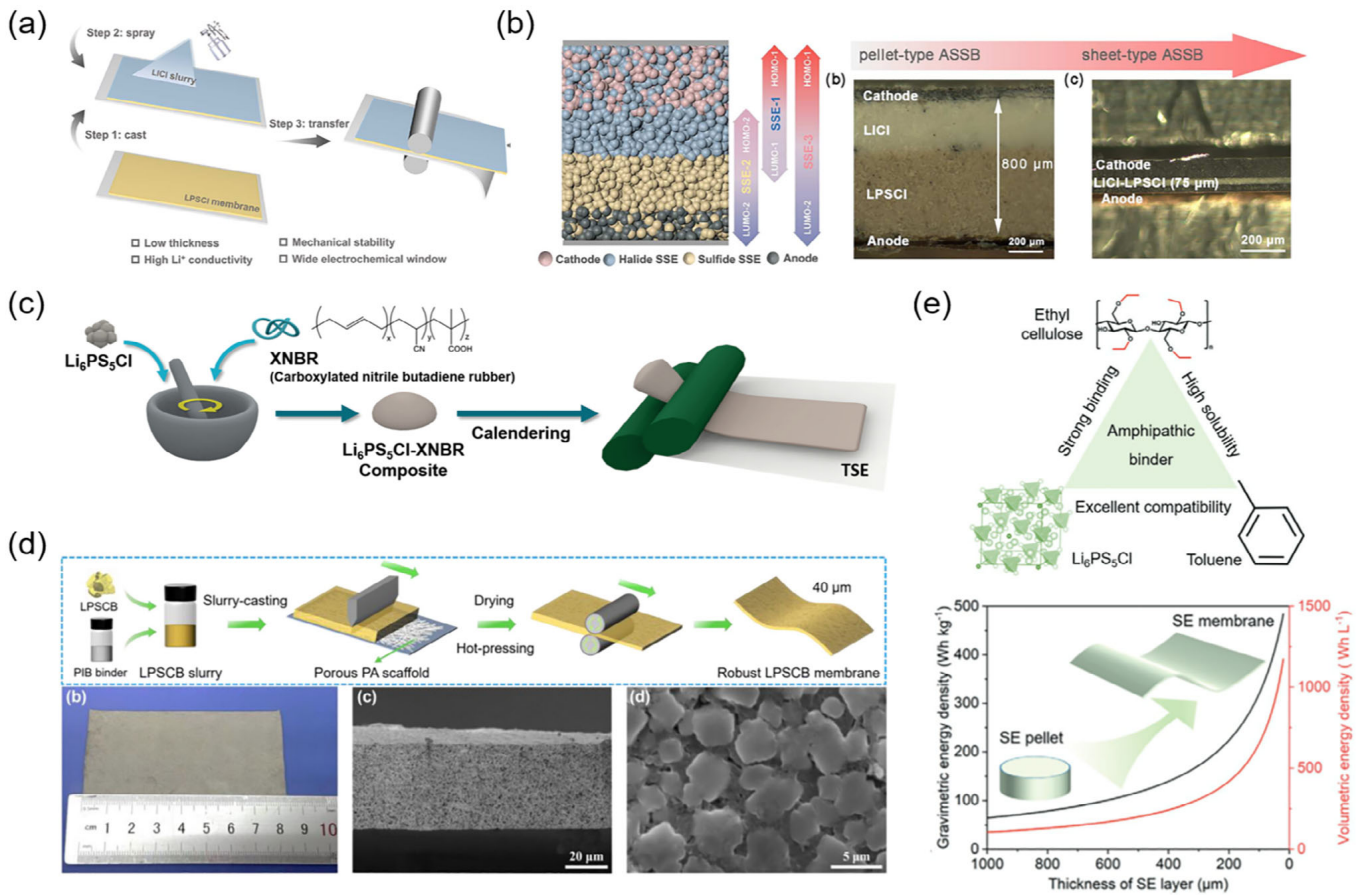
Comparative Wet‐Process Routes for Ultrathin SSE Membranes (a) Schematic of the LICl–LPSCl asymmetrical membrane fabrication process. Reproduced with permission [62]. Copyright 2025, Springer Nature. (b) Schematic diagram of the bilayer electrolyte design to achieve a wide electrochemical stability window. Cross‐sectional images under an optical microscope of ASSBs. Reproduced with permission [62]. Copyright 2025, Springer Nature. (c) Schematic of the TSE separator fabrication process. Reproduced with permission [63]. Copyright 2022, ACS Publications. (d) Schematic illustration of the fabrication process with optical photographs, cross‐sectional, and surface SEM images of the LPSCB membrane. Reproduced with permission [64]. Copyright 2025, Wiley. (e) Illustration of compatibility among LPSCl, ethyl cellulose, and toluene in thin membrane fabrication and estimated gravimetric and volumetric energy densities of ASSBs as a function of the thickness of SSE layers in ASSB coupling LCO and Li metal. Reproduced with permission [57]. Copyright 2021, Wiley.

Figure 4 illustrates four complementary wet‐process routes that advance ultrathin SSE membranes from lab‐scale concepts toward manufacturable, sheet‐type architectures. Jing et al. introduced a spray‐deposited halide capping layer on a cast LPSCl support (Figure 4a), producing an asymmetric LICl–LPSCl laminate that couples chemical robustness at the cathode side with scalable coating/transfer steps [62]. Figure 4b schematically motivates a bilayer solid‐electrolyte design in which a thin halide cap (LICl, ∼10 µm) overlies a sulfide support (LPSCl) so that the low‐voltage stability of halides and the high‐voltage tolerance of sulfides complement each other to broaden the electrochemical stability window, while replacing thick cold‐pressed powder pellets (∼800 µm) with a wet spray‐coating followed by transfer‐printing to produce an asymmetric ∼75 µm membrane that maintains the expanded window and durable cycling [62].

Kim et al. pursued a calendering route in which a carboxylated nitrile butadiene rubber (XNBR) binder and non‐polar solvent build a compliant, processable LPSCl composite thin separator (<50 µm) that cycles under low stack pressure and moderate temperature in Li–S configurations, highlighting the role of viscoelastic binders in mitigating defects during densification (Figure 4c) [63]. Qi et al. combined a PIB binder with a porous polyamide (PA) scaffold to cast and hot‐press a ∼40 µm Li_5.3_PS_4.3_ClBr_0.7_ (LPSCB) film while, in parallel, applying a halide interphase (Li_1.75_ZrCl_4.35_O_0.5_F_0.4_, LZCOF) to Ni‐rich cathodes. This explicit dual‐interface design raises mechanical strength and suppresses interfacial degradation under practical rates (Figure 4d) [64]. Cao et al. developed a filtration pathway using an amphiphilic ethyl‐cellulose binder in toluene to disperse LPSCl and form freestanding, ∼tens‐of‐micrometer films that combine low areal resistance with improved handling; interparticle bridging and binder‐solvent compatibility enable thinning without catastrophic loss of integrity (Figure 4e) [57].

## 4 Dry Process for Sheet‐type ASSB Components

### 4.1 Manufacturing Procedures and Basic Types

The dry process fundamentally differs from the wet process in that it does not require solvents to fabricate ASSBs [65]. Conventional slurry processes disperse binders, active materials, and SSEs in a solvent, coat the slurry onto a current collector, and subsequently remove the solvent through drying. In contrast, the dry process omits slurry preparation and solvent evaporation, significantly reducing process time and energy consumption. In addition, the absence of solvents minimizes environmental concerns and provides substantial cost advantages [66]. In the wet process, chemical compatibility between the SSEs and the solvent or binder must be considered, as reactions can cause electrolyte decomposition or interfacial instability. By contrast, the dry process significantly reduces the risk of such chemical reactions, broadening the range of material selection and enabling process designs that do not compromise electrolyte stability [67]. Furthermore, fabricating thick electrodes in the wet process poses challenges, including controlling slurry viscosity, maintaining dispersion stability, and limiting drying rates. In contrast, the dry process is free from these constraints, making it highly advantageous for high‐loading electrode designs [68]. Owing to these advantages, the dry process has recently emerged as a key strategy for the fabrication of ASSBs and next‐generation batteries, offering competitiveness in process simplification and productivity. To quantify this advantage, it helps to examine the cost structure of the conventional wet route that ASSB slurry processing largely inherits. The most detailed solvent recovery cost models still come from NMP‐based LIB manufacturing. In these models, drying and solvent recovery dominate the energy and capital burdens of electrode fabrication rather than being minor auxiliary steps. Drying and solvent recovery require roughly 10 kWh per kilogram of NMP, which is about 45 times the heat required to vaporize it. The demand is this high because a large airflow must keep the solvent vapor well below its flammability limit. This operation still contributes only a few percent of the total pack cost, around 2.6 to 3.4% across process model‐ and BatPaC (Argonne's BatPaC cost model)‐ based estimates [69, 70]. The dedicated recovery train, comprising a condenser, zeolite wheel, scrubber, distillation column, and chillers, represents about $3 to $6 M of plant capital for a line producing 100,000 packs per year [70]. Recovering the solvent costs about $1.12 per kilogram and saves about $2.08 per kilogram compared with buying fresh NMP at $3.20 per kilogram, yet the recovery train itself remains a large capital expense [69]. These figures bound the conventional NMP case and should not be read as ASSB values. Sulfide‐compatible processing needs low‐polarity solvents such as xylene or toluene rather than NMP. It must also run under dry room or glovebox conditions to preserve electrolyte integrity. Both factors raise the marginal cost of the wet route. Bottom‐up modeling that includes these constraints finds sulfide ASSB cathode processing to be modestly more expensive than its LIB counterpart, at about $4.6 versus $4.1–$4.3 kWh^−1^, mainly because the protective glovebox environment has a shorter depreciation period [35]. Dry processing avoids both the drying ovens and the entire recovery train. Solvent‐free routes are projected to cut process energy by more than 46% and electrode manufacturing costs by roughly 11.5–19% relative to the wet route [71, 72]. This benefit should be larger for sulfide ASSBs given their solvent reactivity and strict atmospheric requirements [35]. These savings, however, do not render dry processing cost‐free. The route remains heavily dependent on PTFE, whose in‐situ fibrillation no alternative binder yet fully reproduces, and fibrillation must be tightly controlled through shear, temperature, and calendering, since excessive fibrillation embrittles the film and degrades mechanical integrity, while high‐loading dry electrodes often trade a measure of rate capability for their thickness [65, 72]. A balanced comparison, therefore, weighs the elimination of the drying oven and recovery train against binder cost, process control, and rate‐performance penalties intrinsic to the dry route. Table 1 summarizes these two routes in terms of solvent use, representative ionic conductivity, favorable thickness range, chemical compatibility, and key limitations. Notably, the representative ionic conductivities of the two routes lie within the same order of magnitude, near 1.12 mS cm^−1^ for the wet tape‐cast film and near 2.3 mS cm^−1^ for the dry film on LPSCl‐based films at room temperature [55, 73], indicating that the choice between them is governed less by intrinsic ionic transport than by the target electrode architecture and the chemical compatibility window.

**TABLE 1:** Comparison of wet and dry processing routes for ASSB fabrication.

Dimension	Wet process	Dry process
Solvent use	Required (low‐polarity aromatics such as p‐xylene or toluene)	Not required
Representative ionic conductivity	1.12 mS cm^−1^ (tape‐cast LPSCl film, 54 wt.% solid loading) [55]	2.3 mS cm^−1^ (LPSCl dry film, substrate‐modulated SBR binder) [73]
Favorable thickness range	Ultrathin SSE membranes, 20 to 80 µm [61]	Thick high‐loading electrodes, 10 to 20 mAh cm^−2^ [79]
Processing cost	Higher due to dry‐room or glovebox requirement	Lower potential; drying oven and solvent‐recovery train eliminated
Chemical compatibility	SSE reactivity with the solvent and binder must be managed	Reaction risk reduced, wider material selection
Key limitation	Solvent and electrolyte reactivity, dry‐room requirement	Dependence on PTFE fibrillation control

**FIGURE 5:**
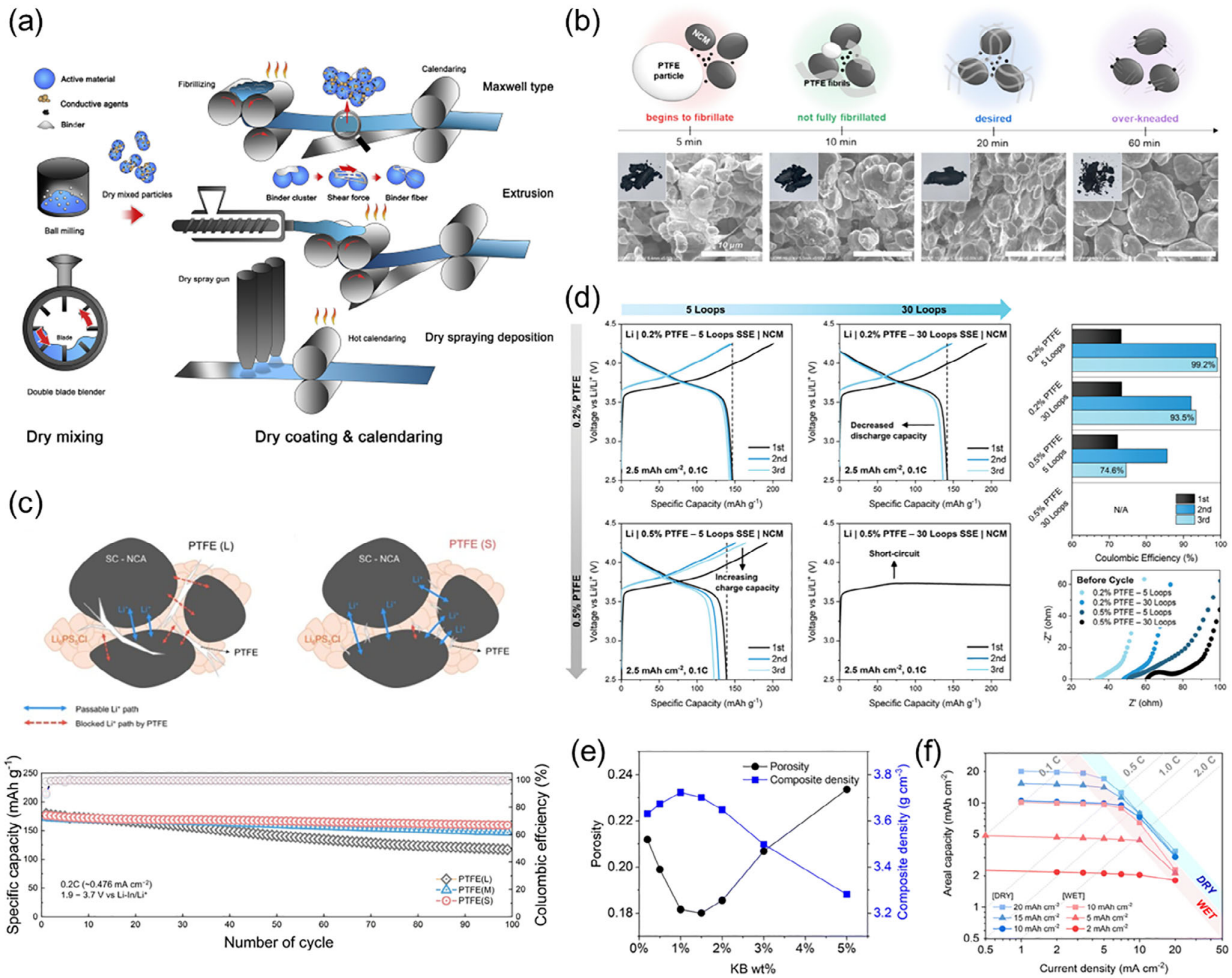
Materials and procedures for the dry process. (a) Typical procedures for applying the dry method for electrode preparation and basic types of dry coating: Maxwell type, extrusion, and dry spraying deposition. Reproduced with permission [66]. Copyright 2022, Elsevier Inc. (b) Schematic diagram showing the fibrillation process of the PTFE binder with kneading time. Reproduced with permission [77]. Copyright 2024, Elsevier. (c) Schematic illustrations of Li^+^ ion movement within the composite cathode adopted with PTFE (L) and PTFE(S), and cyclability test of 100 cycles in ASSBs utilizing PTFE with different sizes. Reproduced with permission [80]. Copyright 2024, Elsevier. (d) Evaluation of cycling stability of SSE films with Li metal anode. Voltage profiles of the first 3 cycles for Li || NCM half‐cell assembled with SSE film. Reproduced with permission [81]. Copyright 2023, Wiley. (e) Porosity of electrodes and half‐cells based on the amount of KB (600JD). Reproduced with permission [79]. Copyright 2024, Royal Society of Chemistry. (f) Discharge rate capabilities of the dry‐processed cathodes with areal capacities of 10, 15, and 20 mA h cm^−2^, respectively. Reproduced with permission [79]. Copyright 2024, Royal Society of Chemistry.

Typically, composites comprising active materials, SSEs, conductive agents, and binders are dry‐mixed to achieve a homogeneous dispersion, then deposited onto current collectors by various methods to form the electrode layer [21]. The binders selected for this process are generally thermoplastic or fibrillating polymers that form a physical network between particles, ensuring the mechanical integrity of the electrode (Figure 5a) [66]. Dry‐coating methods can be classified into several types. The Maxwell method is based on the fibrillation mechanism of the binder, where compression induces fibrous binder networks that connect particles [74]. The extrusion method continuously extrudes the composite mixture through a heated head to fabricate electrodes suitable for large‐area production [75]. In the fine‐particle mixture spray method, coating uniformity and dispersion control are critical, as in the dry‐spraying method [76, 77]. Subsequently, the coated electrodes are densified by calendering to adjust density and improve interfacial contact, then slitted for final cell assembly [66].

Beyond cost, the production rate of electrode sheets is itself a decisive factor for large‐scale ASSB manufacturing, and the two routes differ sharply. In conventional slurry coating, the coating head can run at approximately 20–60 m min^−1^. The effective throughput, however, is set by the downstream drying step rather than by the coating step. The drying stage alone can account for more than 90% of the total electrode manufacturing time [66]. Line speed can therefore rise only by lengthening the already long drying ovens or by adding auxiliary dryers. Maxwell‐type dry coating has instead been reported to reach about 25 m min^−1^ at the research and pilot stage. This rate is comparable to the lower range of slurry coating, yet it removes the drying bottleneck and shifts the rate‐limiting step to powder mixing, binder fibrillation, and calendering [66]. For ASSB electrode sheets in particular, fabrication rates are still reported primarily at laboratory and pilot scales rather than at steady‐state continuous‐line speeds. Coating‐head speeds quoted in m min^−1^ describe linear web advance, whereas the practically required throughput is more meaningfully expressed as an areal coating rate. Zaman and Hatzell estimate that rates on the order of m^2^ min^−1^ are needed for practical implementation, and translating between the two requires the coating width [78]. The moisture sensitivity of sulfide electrolytes adds an additional penalty to the wet route, since drying must be performed under inert or dry room conditions, which constrain throughput. On balance, the dry route gains a structural throughput advantage by avoiding the drying bottleneck, whereas the wet route maintains compatibility with installed coating lines. Realizing the dry‐route advantage at scale will require stable film uniformity and control of binder fibrillation at high line speeds.

### 4.2 Challenges in Key Components of the Dry Process

#### 4.2.1 Polymeric Binders

The successful implementation of dry electrode processing requires a comprehensive understanding of the properties and roles of the key electrode components. In the wet process, solvents and binders assist particle dispersion and adhesion; in the dry process, however, the absence of these auxiliaries means that the physical and chemical properties of the binder, conductive agents, and current collectors directly determine electrode performance. In this context, the binder is pivotal as the material that forms the mechanical interparticle bonding network [53].

Polytetrafluoroethylene (PTFE) exhibits higher chemical stability and lower polarity than PVdF. PTFE can form a fibrous structure that promotes Li^+^ transport and reduces tortuosity [65]. Moreover, during kneading, its intrinsic fibrillation enables the physical intermixing of active materials and conductive agents without the need for solvents, making it highly suitable for dry processing. Oh et al. show the evolution of PTFE fibrillation as a function of kneading time (Figure 5b) [77]. When the kneading time is too short, PTFE is poorly dispersed and forms a weak binder network, resulting in low mechanical stability of the electrode. In contrast, an optimal kneading duration produces long, thin fibrous structures that effectively bind active and conductive particles into a robust polymer network. Excessive kneading, however, can break the fibrous structure, cause agglomeration, and reduce conductivity and increase electrode heterogeneity. Therefore, precise control of PTFE fibrillation conditions is essential in dry electrode fabrication. Figure 5c indicates that reducing the PTFE particle size minimizes binder‐blocked regions and increases the active interfacial area, thereby enhancing ionic/electronic conduction and long‐term kinetics in the composite cathode [80]. Dry composite cathodes with PTFE(S) (cryo‐pulverized, small‐particle) were cycled against PTFE(L) (larger, as‐received); PTFE(S) showed slower resistance growth and ∼90% capacity retention at 100 cycles. Figure 5d demonstrates that higher PTFE content and more calendering loops induce abnormal growth in charge capacity and even immediate shorting, in line with increased cell impedance, highlighting a trade‐off between mechanical strengthening and electrochemical stability in dry‐processed LPSCl films [81].

Although PTFE remains the most widely used binder for dry electrode processing because of its chemical stability and fibrillation capability, its limited adhesion, electrochemical stability, and environmental sustainability have motivated the exploration of alternative dry‐process binders. Beyond PTFE, several dry‐process‐compatible binders and binder systems have been investigated for LIB electrodes and electrolyte films, including CMC/siloxane‐based composites, PP/paraffin wax/stearic acid blends, dry‐powder PVdF, and PEO‐based systems, depending on the processing routes [68, 82, 83, 84]. Similar trends have already been reported in conventional LIB dry electrode manufacturing, where fluorine‐free thermoplastic binders have been investigated to improve adhesion to current collectors and reduce environmental impact. For example, Parafilm M, a paraffin/polyethylene‐based, fluorine‐free thermoplastic binder, has recently been introduced as a dry‐process binder that enables primer‐free adhesion to current collectors and mild‐pressure activation without a wet‐coated primer layer [85]. This approach demonstrated dry NCM811 electrodes with high areal capacities over 5 mAh cm^−2^ during extended cycling, suggesting that fluorine‐free thermoplastic binders can provide a practical route toward more sustainable dry electrode manufacturing. Other binder strategies beyond PTFE, including ionomer‐ and SBR‐based dry‐process binders, are further discussed in relation to functional network design in dry electrodes [73, 86, 87].

#### 4.2.2 Conductive Agents

Conductive agents must be uniformly distributed within the electrode to establish continuous electronic pathways, and this requirement is particularly critical in thick, high‐loading electrodes where no solvent is available to aid dispersion. Kim et al. explained that high‐performance dry electrodes require conductive agents that form a uniform electronic network, lower the percolation threshold, and provide superior mechanical properties for stable electrode structures [65]. Oh et al. fabricated dry electrodes by incorporating porous spherical conductive agents and optimizing their content. Using Ketjen black (KB), which has uniform particle morphology and exhibits a higher electronic percolation network (Figure 5e), they evaluated electrode porosity, composite density, percolation threshold, tortuosity, electrical conductivity, and electrochemical impedance spectroscopy characteristics to determine an optimal content of 2–3 wt.% [79]. In the final electrode design, carbon nanotubes (CNTs) and Super P were combined as conductive agents to facilitate the application of the porous spherical agent. CNTs, with their 1D structure, formed continuous percolation networks, while Super P contributed to surface uniformity and supplemental conductivity, enabling stable electrical and mechanical properties even at low conductive agent contents. The rate performance of electrodes fabricated using this optimized conductive agent ratio is shown in Figure 5f [79]. The dry electrodes maintained high capacities at 0.1–1 C in thick electrodes with areal capacities of 10–20 mAh cm^−2^, demonstrating superior high‐rate performance compared to wet‐processed electrodes and outperforming conventional CNT/graphene‐based dry electrodes.

#### 4.2.3 Functional Network Design in Dry Electrode

**FIGURE 6:**
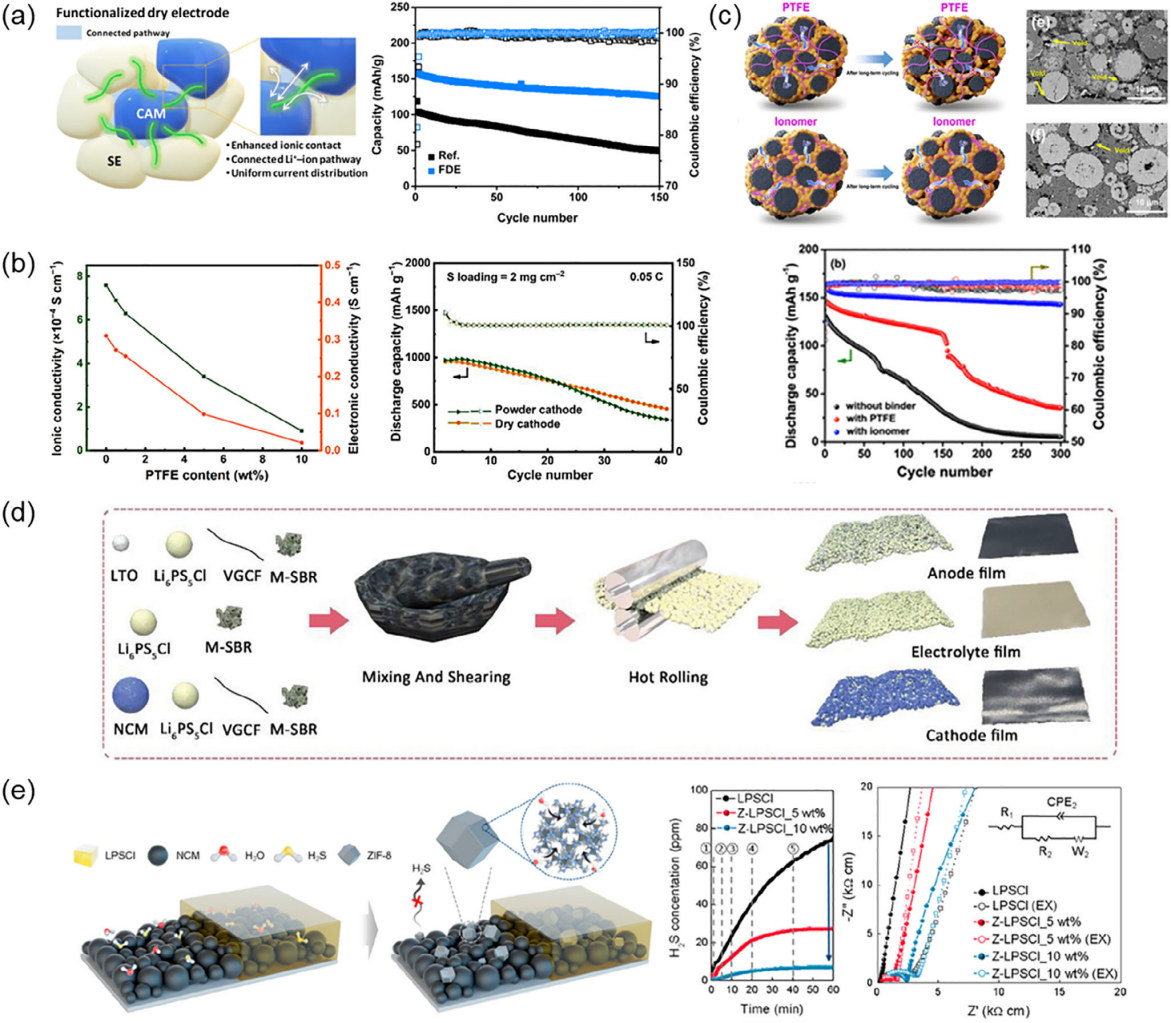
(a) Schematic illustration of  FDE and cycle performance of the cathode with PTFE and FDE at 0.1C at R.T. Reproduced with permission [87]. Copyright 2024, Royal Society of Chemistry. (b) Electronic/ionic conductivities of cold‐pressed powder sulfur cathode and dry‐method produced cathodes with different PTFE contents. Cycling performances of all‐solid‐state Li‐S mold cells using powder and sheet‐type dry cathodes at 0.05 C and 60°C. Reproduced with permission [88]. Copyright 2022, Elsevier. (c) Cross‐sectional SEM images of composite cathodes after 300 cycles with PTFE and with the ionomer, and cycling performance, cycled at 0.5 C and 25°C. Reproduced with permission [86]. Copyright 2022, ACS Publications. (d) Schematic illustration of the preparation process of the cathode, anode, and electrolyte layer of ASSBs. Reproduced with permission [73]. Copyright 2022, Wiley. (e) Schematic illustration showing how ZIF‐8 adsorbs H_2_O, concentration of H_2_S gas in LPSCl and Z‐LPSCl, and Nyquist plots of LPSCl, Z‐LPSCl before and after exposure to moisture. Reproduced with permission [89]. Copyright 2023, ACS Publications.

Beyond individual binders and conductive agents, recent studies have focused on designing integrated functional networks in dry electrodes to simultaneously improve mechanical cohesion, electronic conduction, ionic transport, and environmental stability. Research on the fabrication of dry electrodes for ASSBs has also been actively pursued. The Manthiram group demonstrated that functionalized dry electrodes (FDEs), PTFE‐based dry electrodes incorporating CNT conductive agents and solvate ionic liquid‐infiltrated ethyl cellulose binders, achieved stable electrochemical performance and long cycle life even at areal capacities of approximately 10 mAh cm^−2^ (Figure 6a) [87]. This work highlights the importance of designing integrated binder‐conductive networks in dry electrodes. Hu et al. reported a low‐binder dry processing strategy for thick sulfur cathodes, where fibrous binder networks enabled simultaneous electronic/ionic conduction and mechanical robustness (Figure 6b) [88]. This approach achieved high areal capacities and excellent rate and cycle performance, demonstrating the practical potential of large‐area all‐solid‐state Li–S batteries. Hong et al. developed dry composite cathodes using Li^+^‐conductive ionomer binders, achieving stable ionic and electronic pathways and excellent initial electrochemical performance even in thick electrodes (Figure 6c) [86]. Ionomers, which contain ionic functional groups along their polymer backbone, enhance particle adhesion and mechanical stability while providing Li^+^ conduction. Cross‐sectional scanning electron microscope (SEM) images confirm interfacial contact morphologies with ionomers after 300 cycles. Li et al. introduce a substrate‐modulated dry‐process binder strategy in which SBR is dissolved and reprecipitated on NaCl to tune its packing/dispersion, enabling hot‐rolled cathode, electrolyte, and anode films with improved interparticle adhesion [73]. The processed SBR yields SSE sheets with ∼2.3 mS cm^−1^ ionic conductivity and ASSBs that retain >84% capacity after 600 cycles at 0.3 C, underscoring the viability of dry‐film fabrication beyond PTFE binders (Figure 6d). Jung et al. incorporate zeolitic imidazolate framework‐8 (ZIF‐8) as a moisture‐absorbent additive into LPSCl‐based dry electrodes to passively capture water and suppress moisture‐induced degradation (e.g., H_2_S evolution) without altering the electrode or SE architecture [89]. This simple additive approach stabilizes interfacial impedance and delivers durable cycling of LPSCl ASSBs by maintaining electrolyte integrity under ambient exposure (Figure 6e). These studies demonstrate that dry processing is not limited to replacing slurry casting; it can also be used to engineer functional electrode architectures through binder, conductive agent, and additive design. Based on this solvent‐free concept, dry processing can also be extended from composite electrodes to SSE sheets, as discussed in the following section.

### 4.3 Dry Processing of SSE Sheets

Dry processing has also been applied to the fabrication of SSE sheets, where the main objective is to obtain thin, dense, and mechanically robust electrolyte layers without using processing solvents. This approach is particularly relevant for sulfide SSEs because their chemical sensitivity to polar solvents limits the applicability of conventional wet casting. In dry‐processed SSE sheets, the sheet quality is largely governed by how effectively the fibrillatable binder, typically PTFE, forms a continuous fibrous network that holds the SSE particles together. Yoon et al. systematically investigated the relationship between PTFE fibrillation and dry‐processed LPSCl sheet quality by controlling the shear time, shear temperature, and number‐average molecular weight (Mn) of PTFE [90]. They quantified the degree of PTFE fibrillation using XRD analysis and showed that high‐Mn PTFE reached ≈ 98% fibrillation, forming a highly entangled fibrous network among SSE particles. As a result, a free‐standing LPSCl sheet with a thickness of 18 µm was fabricated using only 0.5 wt.% PTFE, exhibiting an ionic conductivity of 0.85 mS cm^−1^ and an areal conductance of 472 mS cm^−2^. Lee et al. further demonstrated that PTFE fibrillation can be regulated through composite microstructure by incorporating LLZO particles into LPSCl‐based sheets [91]. The harder LLZO particles enhanced interparticle friction and shear‐force transfer during dry processing, leading to a more homogeneous PTFE fibrous network and improved mechanical robustness. The optimized LPSCl/LLZO 10 wt.% sheet enabled an NCM || LPSCl/LLZO 10 wt.% || graphite full cell to retain 81.2% of its capacity after 300 cycles at 0.3C. In addition to thickness reduction and mechanical reinforcement, the electrochemical stability of dry‐processed SSE sheets should be considered, as PTFE‐based binders can undergo reductive degradation at low anode potentials. Rosner et al. analyzed sulfide SSE dry films using electrochemical and surface characterization methods and showed that PTFE content and degree of fibrillation strongly affect interfacial stability [92]. By optimizing the dry‐film processing conditions, they demonstrated long‐term cycling of Si || NMC pouch cells for more than 1300 cycles, highlighting the importance of balancing mechanical robustness with electrochemical stability in PTFE‐based dry SSE sheets. These studies suggest that dry processing of SSE sheets requires simultaneous control of binder fibrillation, microstructural uniformity, mechanical robustness, and interfacial stability.

## 5 Cell Assembly and Configuration

**FIGURE 7:**
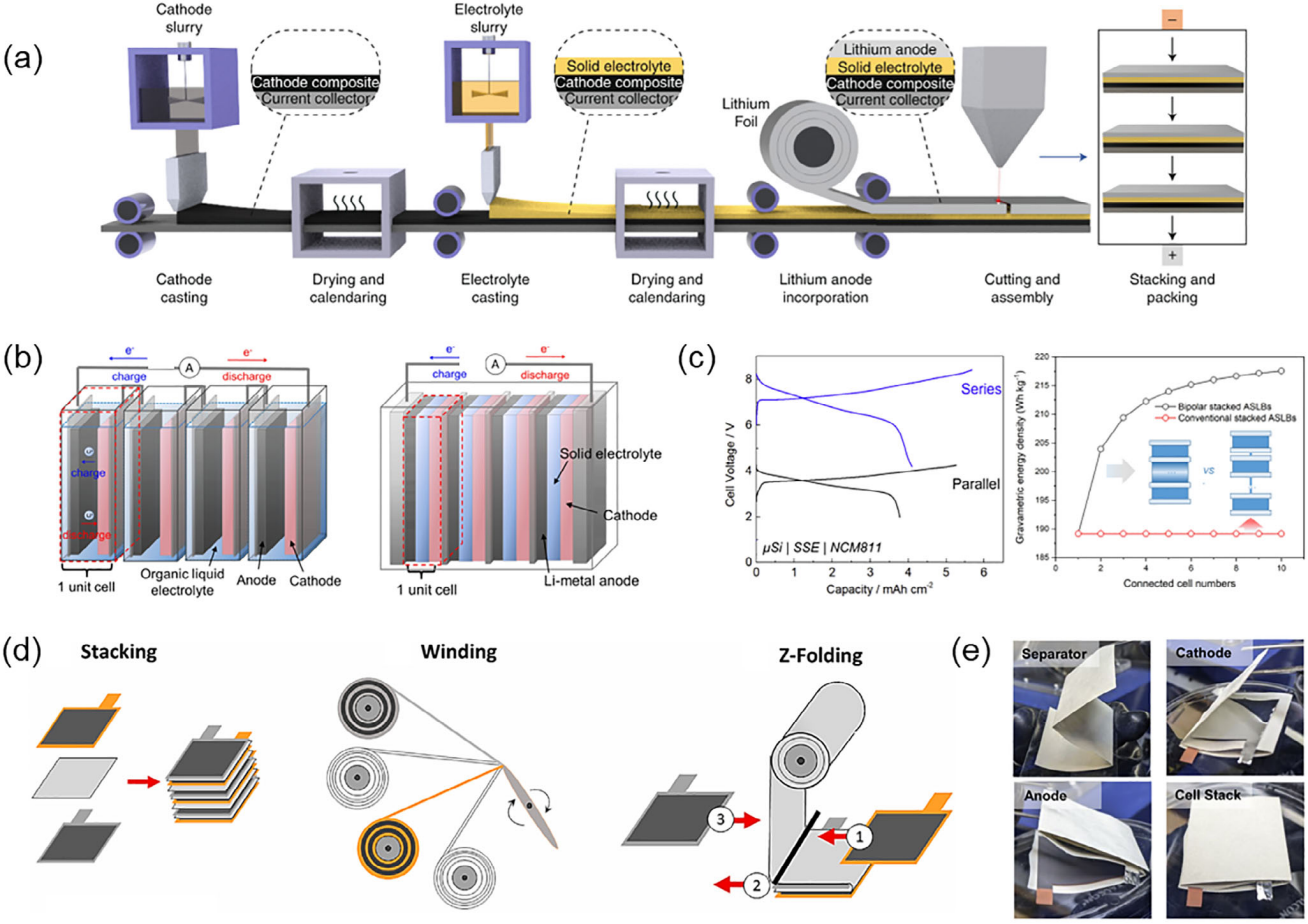
Cell assembly and configurations in ASSB cell manufacturing. (a) Schematic of large‐scale manufacturing of ASSBs. Reproduced with permission [93]. Copyright 2020, Springer Nature. (b) Schematic representation of parallel (bi‐layer) versus series (bi‐polar) stacking design. Reproduced with permission [32]. Copyright 2015, Springer Nature. (c) Benefits of bi‐polar stacking design; comparative voltage profile of parallel‐ versus series‐stacked ASSB design (left) and the gravimetric energy density comparison between the bipolar stacked ASSBs and conventional stacked ASSBs (right). Reproduced with permission [21]. Copyright 2022, Elsevier (left); Reproduced with permission [94]. Copyright 2022, Elsevier (right). (d) Representation of three typical stacking methods: single‐sheet stacking (left), winding (middle), and Z‐folding (right). Reproduced with permission [95]. Copyright 2023, Elsevier. (e) Z‐stacking example using sulfide LPSCl SSE sandwiched between anode and cathode electrode layers. Reproduced with permission [21]. Copyright 2022, Elsevier.

Based on the wet and dry manufacturing technologies for sheet‐type ASSB components discussed earlier, we now turn to examine the cell assembly process where the fabricated electrode and SSE sheets are integrated into full‐cell architectures (Figure 7a) [93]. The selection of cell stacking methods and structures, along with the optimization of pressing processes, goes beyond mere technical completeness; these are critical factors that directly impact battery performance, manufacturing efficiency, and economic viability. Therefore, understanding and optimizing cell‐level assembly technologies is essential for translating processed ASSB sheets into practical battery cells.

### 5.1 Cell Stacking

Depending on the electrode fabrication process, methods for constructing individual stacks can be broadly classified into two approaches. The first is the cathode‐supported two‐layer method, in which the SSE layer is cast directly onto the cathode layer, followed by anode‐layer stacking. The second is the individual three‐layer method, in which the cathode, SSE, and anode layers are manufactured separately and then stacked sequentially [43]. ASSBs achieve high capacity by stacking these individual stacks in multiple layers. The key differentiating factor of ASSBs from conventional LIBs is the possibility of implementing bipolar stacking structures, as illustrated in Figure 7b [33]. Conventional LIBs adopt a bilayer design in which each current collector serves exclusively as either the cathode or the anode. Furthermore, because liquid electrolytes are fluid, each stack must be individually packaged, and multiple cells must be connected in series via external wiring. In contrast, bipolar cells feature multiple unit cells connected in series via bipolar current collectors, in which a single collector simultaneously serves as the anode of one cell and the cathode of an adjacent cell [96, 97]. One side of the current collector is coated with cathode material. In contrast, the other side is coated with an anode material, forming a structure in which all unit cells are connected in series via a single current collector, with no external connections.

The most remarkable advantages of bipolar cells are the substantial improvement in energy density and their suitability for high‐voltage operation [98, 99]. First, regarding high‐voltage implementation, as demonstrated in the mSi | SSE | NCM811 cell configuration example in Figure 7c, higher cell voltage can be achieved through a series connection within a single package [21]. This structure has the potential to reduce voltage ramping requirements in high‐voltage devices. Meanwhile, in terms of energy density, volumetric energy density increases because the number of unnecessary packaging materials and electrical connection components can be significantly reduced. Cao et al. introduced a bipolar stacking design for sulfide‐based ASSBs and reported an increase in energy density from 189 to 217 Wh kg^−1^ with increasing connected cell numbers, as shown in Figure 7c [94].

ASSBs require pressing processes after cell fabrication, making it highly likely that pressing‐compatible stacking‐type assembly methods will be adopted; details will be discussed later. Whereas conventional LIBs employ cell assembly methods such as individual sheet stacking, Z‐folding, or winding, depending on whether the cells are prismatic or cylindrical, as depicted in Figure 7d [95], ASSBs are likely to adopt pouch‐type formats that facilitate the application of stacking pressure. Consequently, Z‐folding has emerged as a particularly noteworthy technology for implementing bipolar stacking [100]. Z‐folding is an assembly technique that involves folding an extended freestanding solid electrolyte separator in a zigzag pattern to arrange cathode and anode electrode layers. Figure 7e demonstrates this assembly method, which not only fundamentally prevents direct contact between the cathode and anode but also offers the practical advantage of enabling mass production with commercial automated equipment [21].

### 5.2 Pressure Strategies for Cell Manufacturing

**FIGURE 8:**
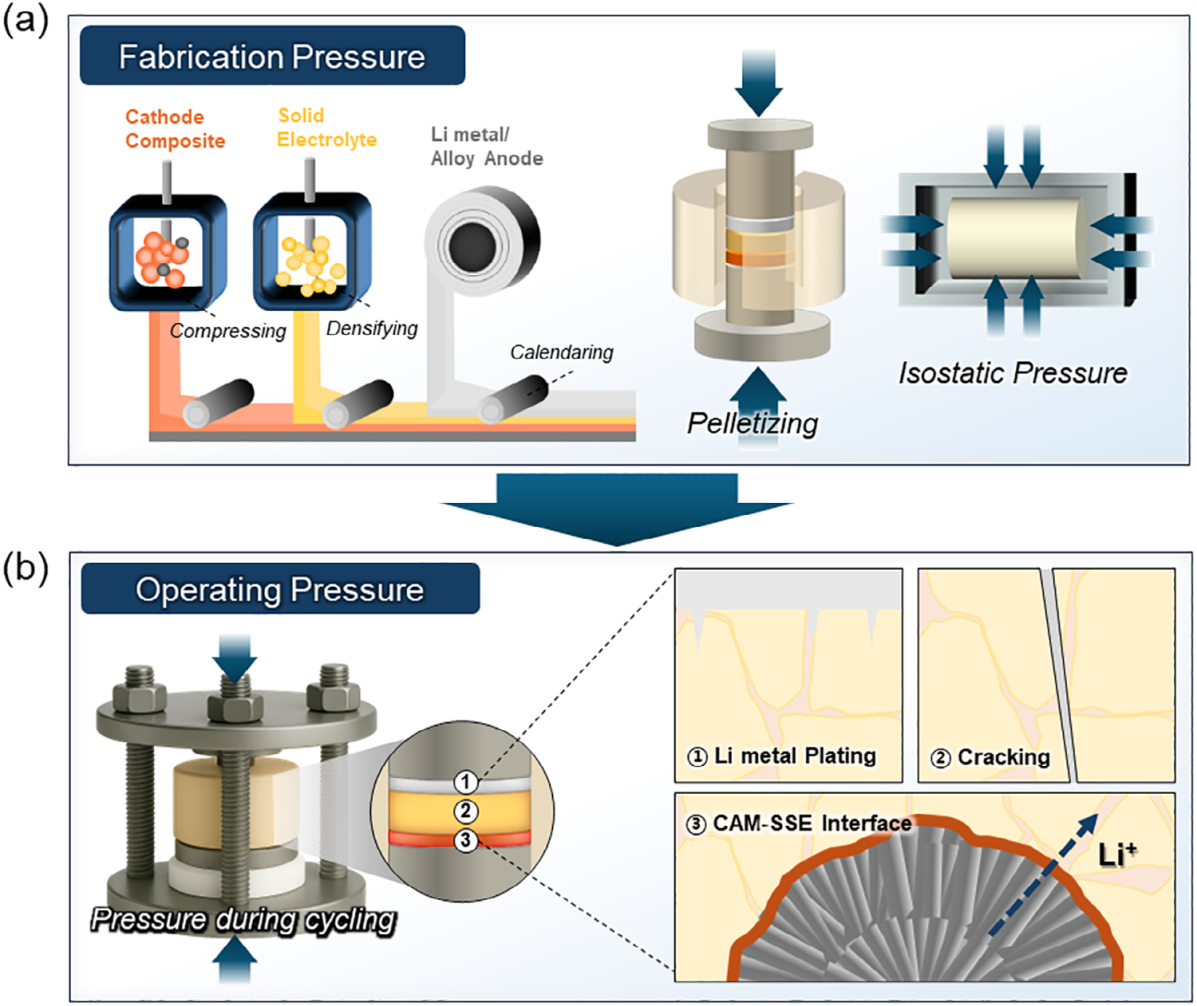
Schematic of the categories and origins of pressure‐related challenges of ASSBs. (a) Fabrication pressure applied externally during compression, densification, and calendering. (b) Operation pressure externally applied during battery cycling.

In ASSBs, pressurization is essential for enhancing interfacial contact between electrodes and SSE layers, improving ionic conductivity, and ensuring stable cycle performance [100, 101, 102, 103]. The pressing process in ASSBs is broadly categorized into fabrication and operating pressure, and understanding their respective impacts on battery performance is crucial (Figure 8).

**FIGURE 9:**
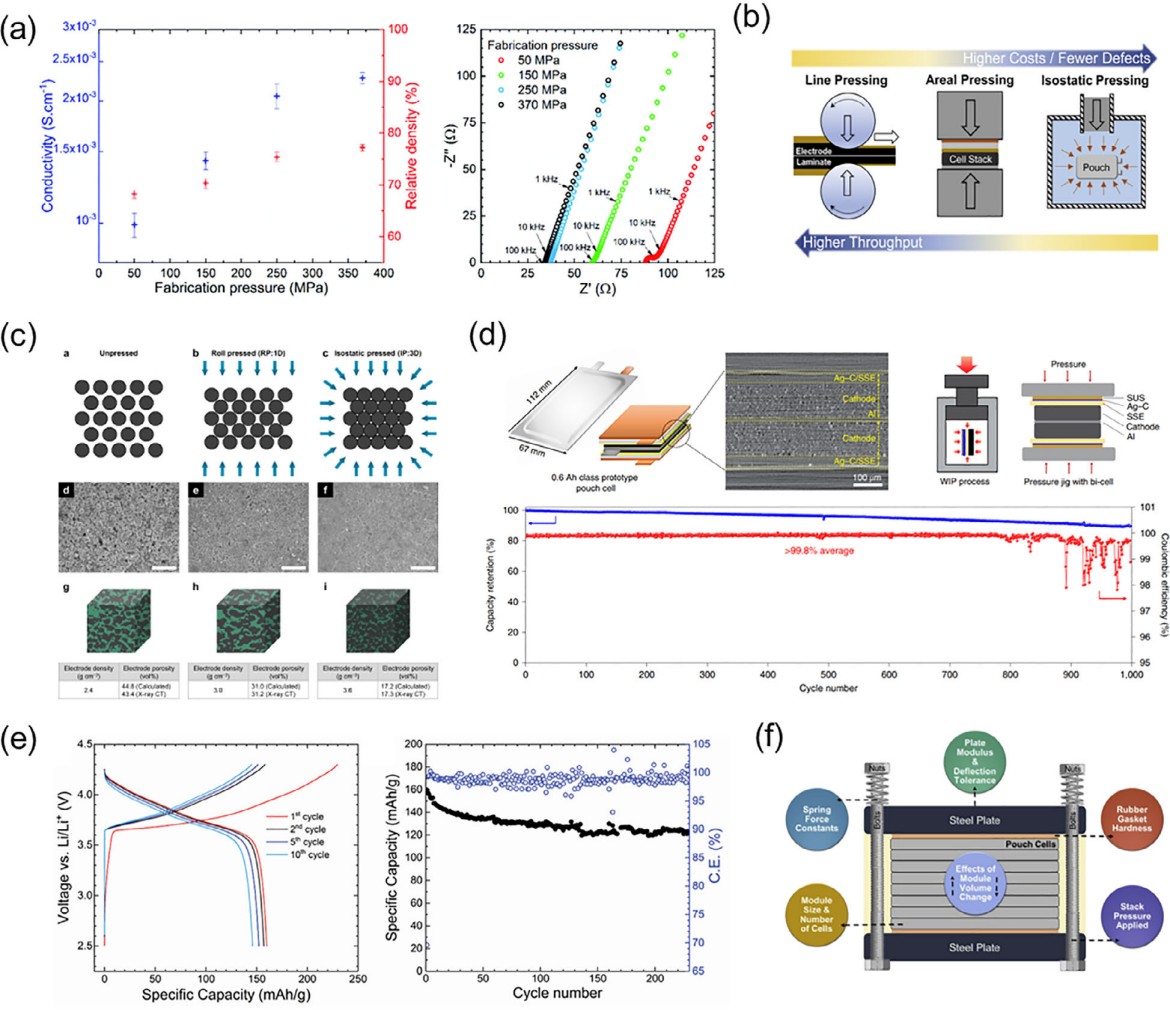
Pressure Strategies for Cell Manufacturing and Testing in ASSBs. (a) Correlation between fabrication pressure and the resulting conductivity/density properties of the LPSCl electrolyte (left) and corresponding Nyquist plots (right). Reproduced with permission [26]. Copyright 2020, Royal Society of Chemistry. (b) Graphical representation of three primary techniques for densification of stacks in pouch‐type cells. Reproduced with permission [21]. Copyright 2022, Elsevier. (c) Schematic diagram, SEM images, and X‐ray CT images showing the microstructures obtained using different pressing techniques. Reproduced with permission [104]. Copyright 2024, Springer Nature. (d) Characterization and electrochemical performance of Ag–C | SSE | NMC prototype pouch cell (0.6 Ah) and schematic of pressurization process during the fabrication and operation of ASSB. Reproduced with permission [105]. Copyright 2020, Springer Nature. (e) Performance of Li | LPSCl | LNO‐coated NCA full‐cell cycled at C/10 under 5 MPa fabrication pressure. Reproduced with permission [28]. Copyright 2020,  Wiley. (f) Consideration factors for ASSB module assembly. Reproduced with permission [21]. Copyright 2022, Elsevier.

Fabrication pressure involves densification, in which stacked cell layers are pressed to reduce porosity and enhance interfacial contact between the electrodes and the SSE. Doux et al. quantitatively evaluated the effect of fabrication pressure on electrochemical performance. They reported that for LPSCl electrolyte, increasing the fabrication pressure from 50 to 250 MPa significantly increased ionic conductivity from 0.99 to 2.06 mS cm^−1^. This is attributed to improved interparticle contact and the formation of an effective ionic conduction pathway via pore‐structure densification, as shown in Figure 9a [26]. The pressing methods for applying such fabrication pressure can be classified into three types: continuous line pressing, uniaxial pressing, and isostatic pressing [21]. Figure 9b illustrates the three primary techniques for densifying stacks in pouch‐type cells. Continuous line pressing, commonly used in LIBs, provides high throughput but can cause non‐uniformity and mechanical cracking issues when applied to ASSBs due to high pressures exceeding 300 MPa. Uniaxial pressing is currently the most dominant method used in pellet‐cell‐type ASSBs and is effective at the laboratory scale. However, it has limitations when applied to large cells as the required pressure increases proportionally with area. Isostatic pressing can produce high‐density electrode forms by applying uniform pressure in all directions and is considered the most effective method, even with minimal cell size constraints.

Lee et al. reported significant differences in the actual microstructural changes observed across pressing methods [104]. Figure 9c presents schematic diagrams, SEM images, and X‐ray computed tomography (CT) images illustrating the microstructures obtained with different pressing techniques. Unpressed electrodes exhibited a high porosity of 44.8 *vol*.% and a low density of 2.4 g cm^−3^. Roll pressing reduced porosity to 31.0 *vol*.% and improved density to 3.0 g cm^−3^, but caused vertical expansion due to unidirectional compression. In contrast, isostatic pressing achieved the lowest porosity of 17.2 *vol*.% and the highest density of 3.6 g cm^−3^, demonstrating the best densification. X‐ray CT analysis confirmed that calculated and measured values matched, demonstrating that isostatic pressing improves electrode/SSE layer interfacial contact through uniform three‐dimensional compression.

WIP (warm isostatic pressing) has recently emerged as a key technology. WIP densifies materials by applying pressures up to 600 MPa, producing high‐density electrode forms through uniform pressure in all directions. The WIP process shows improvements in porosity, adhesion, and rate performance compared to conventional manufacturing methods in multilayer pouch cell processing, and is recognized as a core technology for realizing next‐generation ASSBs. Notably, research from SAIT (Samsung Advanced Institute of Technology) achieved energy densities exceeding 900 Wh L^−1^ and excellent cycle performance over 1,000 cycles in Ag‐C|SSE|NMC prototype pouch cells (0.6 Ah) by applying 490 MPa pressure using WIP, as presented in Figure 9d [105]. Meanwhile, operating pressure is the pressure applied during ASSB operation, which maintains continuous contact between the electrodes and the electrolytes and prevents delamination caused by volume changes during charge‐discharge processes [27, 106]. Doux et al. demonstrated stable room‐temperature cycling of a Li | LPSCl | LNO‐coated NCA cell at an optimized stack pressure of 5 MPa. The cell achieved over 200 cycles, retaining 80.9% of its capacity after 100 cycles with an average Coulombic efficiency of 98.86%. This result highlights that appropriate pressure control is critical for suppressing lithium dendrite formation and ensuring long‐term cycling stability in all‐solid‐state lithium metal batteries (Figure 9e) [28]. Cell‐level design considerations should also account for future module integration requirements. Such integration requires consistent mechanical properties and pressure‐distribution characteristics to accommodate uniform stack pressure across larger cell formats, as depicted in Figure 9f [21].

### 5.3 Low‐Pressure Operation

The optimization of appropriate fabrication and operating pressures, as discussed above, plays a crucial role in enhancing the electrochemical performance of ASSBs. However, the high‐pressure conditions of several tens of MPa demonstrated at laboratory scale pose significant constraints for practical industrial implementation [111, 112], including challenges in large‐area cell manufacturing, increased complexity in pack design, and additional costs associated with pressure application systems. For commercialization in large‐scale applications such as electric vehicles, stable battery operation must be achievable at low pressures below 2 MPa, or ideally under pressure‐free conditions. This requirement represents one of the most pressing challenges facing current ASSB technology. Against this backdrop, recent research has focused on innovative material design, interface engineering, and cell‐structure optimization to enable low‐pressure operation while maintaining excellent performance [113].

**FIGURE 10:**
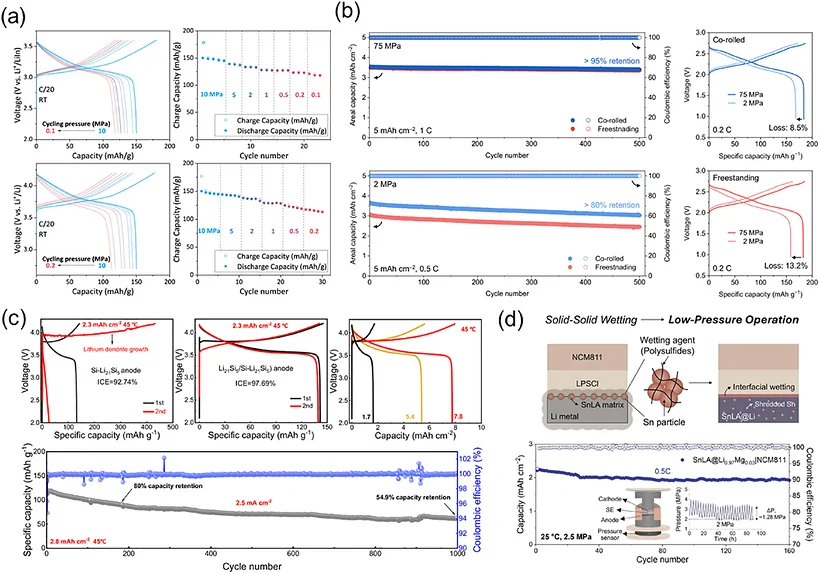
Strategies for low‐pressure operation in ASSBs. (a) Performance of LYBC‐based cells at varying pressures: LiIn anode (top) and Li metal anode (bottom). Reproduced with permission [107]. Copyright 2024, ACS Publications. (b) Capacity retention of co‐rolled cathode films at 2 MPa compared to freestanding films. Reproduced with permission [108]. Copyright 2025, Springer Nature. (c) Li_21_Si_5_/Si‐Li_21_Si_5_ double‐layered anode performance under pressure‐free operation. Reproduced with permission [109]. Copyright 2025, Springer Nature. (d) Long‐term cycling stability at 2.5 MPa using polysulfide‐mediated interfacial wetting. Reproduced with permission [110]. Copyright 2025, Elsevier.

Enhancing interface stability has emerged as a key strategy for achieving low‐pressure operation. Hennequart et al. demonstrated stable operation even at 0.1 MPa (atmospheric pressure) using a halide‐based Li_3_YBr_2_Cl_4_ (LYBC) electrolyte, providing an essential breakthrough for the practical implementation of ASSBs [107]. As shown in Figure 10a, cells operated reliably down to 0.1 MPa (LiIn) and 0.2 MPa (Li metal) at room temperature. This low‐pressure operation stems from the inherently low hardness of the halide electrolyte. Being substantially softer than typical sulfide or oxide electrolytes, it deforms readily and maintains intimate contact with the cathode active material. Lee et al. achieved low‐pressure operation at 2 MPa through a co‐rolling dry process that creates robust SSE‐electrode interfaces during fabrication. This interface minimized void formation under low pressure, enabling over 80% capacity retention after 500 cycles at 2 MPa. In contrast, freestanding films exhibited severe degradation at the same pressure due to interfacial voids, as confirmed in Figure 10b [108].

Meanwhile, innovative approaches for low‐pressure operation are also being pursued on the anode side [114, 115]. While ASSBs offer potential for high‐capacity silicon anodes, their practical application has been hindered by silicon's significant volume changes, which traditionally demand high external pressure. Zhang et al. addressed this by developing a Li_21_Si_5_/Si‐Li_21_Si_5_ double‐layered anode that enables pressure‐free operation and requires only a minimal initial load of 0.8 MPa. Fabricated via cold‐pressed sintering, this anode design promotes a uniform electric field at the anode‐SSE interface and efficiently disperses volume‐expansion stresses within the silicon. As shown in Figure 10c, this advancement led to a high initial Coulombic efficiency of 97 ± 0.7% (at 45°C) and an impressively low expansion rate of 14.5% after 1000 cycles. These results effectively remove the need for conventional high stack pressures [109]. Whereas the silicon‐anode strategy mitigates bulk volume change, lithium‐metal anodes present a different obstacle to low‐pressure operation. Poor contact between Li and the SSE leads to interfacial instability and dendritic growth. Je et al. addressed these issues by introducing a polysulfide‐mediated solid‐state wetting strategy [110]. Their approach uses α‐lipoic acid (LA)‐derived polysulfides and lithiophilic tin (Sn) seeds to form robust interfacial bridges between the Li metal anode and the SSE. This process also drives the depth‐wise redistribution of pulverized Sn seeds, enabling comprehensive wetting across the anode structure. This architecture enables stable operation at room temperature and a low stack pressure of 2.5 MPa. As demonstrated in Figure 10d, the resulting cell achieves exceptional capacity retention of 86.7% over 160 cycles at 0.5C, with a high areal capacity of 3.5 mAh cm^−2^ [110].

Taken together, these strategies show that low‐pressure operation can be approached from three complementary directions: electrolyte selection, electrode‐electrolyte interface construction, and anode architecture. Each direction carries its own trade‐offs, as summarized in Table 2. Halide‐electrolyte cells reach the lowest operating pressures of 0.1 to 0.2 MPa owing to the intrinsic softness of the electrolyte. Their reductive instability against Li metal and comparatively modest ionic conductivity, however, constrain practical energy output. Interface‐oriented routes, namely the co‐rolling dry process and polysulfide‐mediated wetting, sustain stable cycling at 2 to 2.5 MPa, delivering high areal capacities of up to 5 mAh cm^−^^2^ by suppressing interfacial void formation. These routes still depend on the long‐term durability of the binder or interphase. The double‐layered silicon anode nearly eliminates the stack‐pressure requirement at 0.8 MPa by accommodating internal volume change, although it requires an elevated operating temperature and limited areal loading. This comparison indicates that no single strategy simultaneously meets the pressure, energy density, and durability targets required for commercialization. Hybrid approaches that couple soft and electrochemically stable electrolytes with pre‐engineered low‐void interfaces and stress‐tolerant electrode architectures will likely be necessary to achieve sub‐2 MPa or pressure‐free operation without sacrificing performance.

**TABLE 2:** Comparison of representative low‐pressure strategies for ASSBs.

Strategy (approach)	Operating condition	Demonstrated performance	Key limitation
Halide electrolyte – Hennequart et al. [107]	0.1 MPa (LiIn) / 0.2 MPa (Li metal), RT	Stable down to 0.1 MPa; ∼145 mAh g^−1^ at high loading (3.7 mAh cm^−2^)	Reductive instability against Li metal; low ionic conductivity (1.27 mS cm^−1^)
Co‐rolling dry process – Lee et al. [108]	2 MPa, RT	>80% retention, 500 cycles (5 mAh cm^−2^)	PTFE‐binder reduction on extended cycling
Double‐layered Si anode – Zhang et al. [109]	0.8 MPa, 45°C	97 ± 0.7% ICE; expansion suppressed to 14.5% after 1000 cycles	Requires 45°C and high N/P (low loading)
Polysulfide interfacial wetting – Je et al. [110]	2.5 MPa, RT	86.7% retention, 160 cycles (0.5C); 3.5 mAh cm^−2^	The highest stack pressure among these strategies

## 6 Conclusion and Outlook

ASSBs are expected to be game changers in the energy storage market, offering next‐generation systems with enhanced safety and significantly higher energy densities than conventional lithium‐ion batteries. This review systematically examines the comprehensive electrode and cell manufacturing processes essential to realizing the commercial potential of ASSB technology.

In current ASSB manufacturing systems, different fabrication processes employ distinct processing strategies. Wet processing primarily relies on solvent‐based slurry casting methods similar to those used in conventional LIB production, whereas dry processing eliminates solvents through mechanical fibrillation and thermal bonding. Despite variations in processing routes, these methods share common objectives: achieving intimate solid‐solid contact between electrodes and electrolytes, minimizing porosity and tortuosity to enhance ionic transport, and establishing mechanically robust architectures that withstand volume changes during cycling.

Wet processing leverages existing LIB infrastructure for cost‐effectiveness and can produce ultrathin solid‐electrolyte membranes (20–80 µm), which are essential for achieving competitive energy densities. Identifying low‐polarity aromatic solvents and ionically conductive binders addresses chemical compatibility challenges with sulfide electrolytes. Although wet processing may raise concerns about solvent‐electrolyte reactivity, it requires minimal modification to existing production lines and allows direct adaptation of established coating technologies. Conversely, dry processing eliminates solvent use for reduced manufacturing steps and environmental benefits, while proving advantageous for fabricating thick electrodes with high areal capacities (10–20 mAh cm^−2^). Fibrillating polymer‐based mechanisms create mechanically robust electrodes, with recent advances incorporating ionically conductive binders and moisture‐scavenging additives expanding capabilities beyond conventional systems.

Cell assembly strategies emphasize bipolar stacking to enhance energy density and the use of Z‐folding technology for scalable production. Pressure optimization through WIP achieves energy densities exceeding 900 Wh L^−1^ and extended cycle life over 1000 cycles. Recent advances in low‐pressure operation demonstrate significant progress toward practical implementation.

Looking ahead, several key directions merit further investigation to advance ASSB manufacturing technology:

1. Current studies on wet processing predominantly focus on solvent‐binder optimization, while challenges persist in achieving uniform thin‐film solid electrolyte membranes over large areas. Future research should emphasize precise control of slurry rheology, dispersion stability, and drying kinetics to ensure defect‐free films with consistent ionic conductivity across commercially relevant scales. Building quantitative film‐quality assessments such as the X‐DLVO analysis discussed above, coupling continuous coating lines with in‐line thickness and defect monitoring would enable closed‐loop correction during production rather than post‐hoc verification. Such control becomes increasingly important as the field targets ever‐thinner membranes over large areas to achieve competitive energy densities.
2. The interfacial stability between electrodes and solid electrolytes during prolonged cycling remains a critical challenge. Further investigation is needed into advanced interfacial engineering strategies, including protective coatings, buffer layers, and ionically conductive interfacial phases that can maintain low impedance and prevent degradation under practical operating conditions. These designs should be guided by in‐depth characterization, such as X‐ray computed tomography and impedance analysis used throughout this review, which identifies where degradation begins, thereby making the interface engineering mechanism‐driven rather than empirical. Establishing quantitative links between interfacial impedance growth and microstructural change would further turn these observations into transferable design rules.
3. The low‐pressure operation strategies show great potential, and their industrial implementation should be accelerated. By integrating interface engineering, structural design optimization, and advanced electrolyte materials, the manufacturing process can achieve operational pressures below 2 MPa, significantly enhancing commercial feasibility and reducing system‐level complexity required for pressure maintenance. As Section 5.3 shows, each existing strategy addresses only one origin of pressure dependence. Combining soft electrolytes, low‐void interfaces, and approaches that mitigate anode volume change is therefore more promising than optimizing any single factor. Reporting stack pressure alongside areal loading and temperature in a standardized way would also let the field benchmark these strategies consistently.
4. Scaling from laboratory‐scale to large‐format commercial cells presents significant manufacturing challenges. Densification methods such as warm isostatic pressing, which have enabled high‐energy‐density prototype pouch cells, should be systematically translated to multilayer pouch and prismatic formats, with attention to how pressure uniformity and yield evolve as cell area and stack count increase. Non‐destructive inspection and statistical process control would help minimize cell‐to‐cell variability in solid‐state architectures before defects propagate to the module level. Co‐designing cell format, bipolar architecture, and module‐level pressure hardware from the outset will be essential to carry laboratory performance into manufacturable, EV‐scale products.

## Conflicts of Interest

The authors declare no conflicts of interest.

## Data Availability

Data sharing not applicable to this article as no datasets were generated or analyzed during the current study.
